# A Mobile Acoustic Subsurface Sensing (MASS) System for Rapid Roadway Assessment

**DOI:** 10.3390/s130505881

**Published:** 2013-05-08

**Authors:** Yifeng Lu, Yi Zhang, Yinghong Cao, J. Gregory McDaniel, Ming L. Wang

**Affiliations:** 1 Department of Civil and Environmental Engineering, Northeastern University, 360 Huntington Avenue, Boston, MA 02115, USA; E-Mails: rhyzhang@gmail.com (Y.Z.); yingh.cao@gmail.com (Y.C.); Mi.Wang@neu.edu (M.L.W.); 2 Department of Mechanical Engineering, Boston University, 110 Cummington Street, Boston, MA 02215, USA; E-Mail: jgm@bu.edu

**Keywords:** mobile, acoustic, subsurface, MASS, radiating surface wave

## Abstract

Surface waves are commonly used for vibration-based nondestructive testing for infrastructure. Spectral Analysis of Surface Waves (SASW) has been used to detect subsurface properties for geologic inspections. Recently, efforts were made to scale down these subsurface detection approaches to see how they perform on small-scale structures such as concrete slabs and pavements. Additional efforts have been made to replace the traditional surface-mounted transducers with non-contact acoustic transducers. Though some success has been achieved, most of these new approaches are inefficient because they require point-to-point measurements or off-line signal analysis. This article introduces a Mobile Acoustic Subsurface Sensing system as MASS, which is an improved surface wave based implementation for measuring the subsurface profile of roadways. The compact MASS system is a 3-wheeled cart outfitted with an electromagnetic impact source, distance register, non-contact acoustic sensors and data acquisition/processing equipment. The key advantage of the MASS system is the capability to collect measurements continuously at walking speed in an automatic way. The fast scan and real-time analysis advantages are based upon the non-contact acoustic sensing and fast air-coupled surface wave analysis program. This integration of hardware and software makes the MASS system an efficient mobile prototype for the field test.

## 1. Introduction

The highway pavement and bridge deck health situation is critical for our society. It affects the safety of millions of people daily and requires financial investments that benefit from detailed knowledge. Pavement and bridge deck health diagnosis has become very important as the traffic efficiency and personal security should be protected. The conventional pavement and bridge deck inspection technologies are designed to diagnose surface defects such as different sizes of cracks, potholes, and roughness, since it is quite easy to visually inspect the surface. However, the deterioration of pavement initiates not only from surface cracks and potholes due to tire scratching, but also from the de-bonding of subsurface layers, or stripping due to material aging and corrosion. Defects caused by subsurface damage often develop before the surface defects are visible.

For the inspections of the subsurface of pavement and bridge roadway, many methods have been developed and are being utilized for fieldwork testing. Impact Echo (IE), Impulse Response (IR), Ground Penetrating Radar (GPR), Chain-Drag, and Spectral Analysis of Surface Waves (SASW) are typical technologies. IE is limited to identifying the de-bonding and thickness of a shallow top layer [1]. IR could test the overall dynamic stiffness/mobility of the entire pavement structure [2]. GPR is best for locating the metal materials, such as reinforcement rebar [3]. Chain-drag could find the de-bonded or corroded areas by hearing the resulting hollow sound with expert experience [4]. The SASW and its related methods are frequently used as their capability of estimating the thickness and stiffness of subsurface layers [5–7].

**Figure 1.**
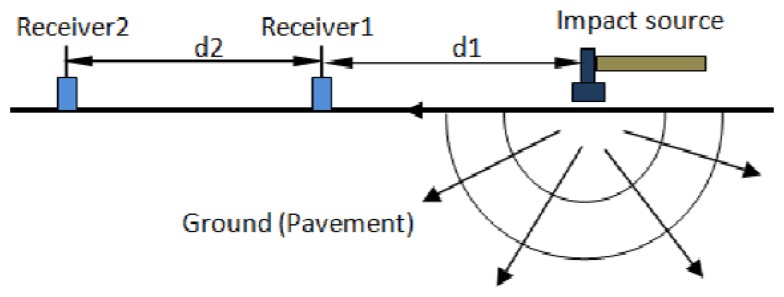
Test configuration of SASW Method.

Since it was first proposed in the 1980s [5], the SASW method has been widely applied in geology field tests to estimate the underground soil profile without coring or opening the ground [8–10]. Figure 1 shows representative experimental layout for implementing the method. It utilizes the dispersion features of the surface wave that propagates horizontally in the soil when it is subject to an impact load. The dispersion curve represents the relationship between the wave speed, wave length and frequency. It is measured by phase difference between two receivers with known distance. Once the dispersion curve is obtained from the test data, the layer profile as shear velocities can be estimated by inversion procedures. Extensive research has been conducted to improve the accuracy and efficiency of the method. For the inversion procedure, analytical forward modeling for dispersion curve is needed. One typical method is the stiffness matrix method [11], which predicts the analytical dispersion curve used as the matching object for an inversion iteration. In recent years, the SASW method was extended to investigate shallow layered structures such as pavement systems [12,13] and concrete structures [14]. Some similar methods were successfully developed based on the same principle of SASW. For example, the Multichannel Analysis of Surface Wave method uses multiple sensors to record the complete wave field and resolves the different wave modes [15].

Despite these past achievements of SASW and related methods, the methods are labor intensive in two ways. Firstly, the method requires the installation and repositioning of ground-mounted sensors to measure the ground vibration, such as accelerometers and geophones. Secondly, the inversion algorithms often require human adjustments based on experience. Another critical drawback is the disruption to traffic flow due to the requirement of attaching sensors to the ground. Recently, attempts were made to improve the testing efficiency by using noncontact sensors, such as microphones, to replace the traditional contact accelerometers [16], in which the surface wave measured by the microphones is used to obtain the dispersion feature. A mobile testing concept was also investigated which could perform the fast scanning on concrete plates successfully [17].

This paper presents an integrated mobile acoustic sensing system which was developed to estimate the thickness and elastic modulus for pavement layers at walking speed. An electrically powered hammer is used to produce an impact force to the ground. A microphone array is used to collect acoustic signals radiated from the surface wave. Multi-channel DAQ is assembled to collect and process data with a laptop computer. An encoder-based register locates the distance along the scanning path and triggers the hammer impact with the desired scan resolution. A key feature of this prototype is its real-time display of subsurface properties. To achieve this, the inversion procedure in air-coupled SASW is conducted with a fast inversion algorithm. This algorithm connects the dispersion curve with layered shear velocity profile directly by the *in-situ* particle displacement distribution [18]. The benefit of the algorithm is the avoidance of the need for expertise knowledge to initialize the forward profile model as well as trial and error iteration. More details of the algorithm are introduced by [18]. The prototype was tested at several fields, including the asphalt pavement and bridge deck. The test results demonstrate the concept and capability of the MASS prototype.

## 2. Physics behind MASS System

### 2.1. Air-Coupled SASW

**Figure 2.**
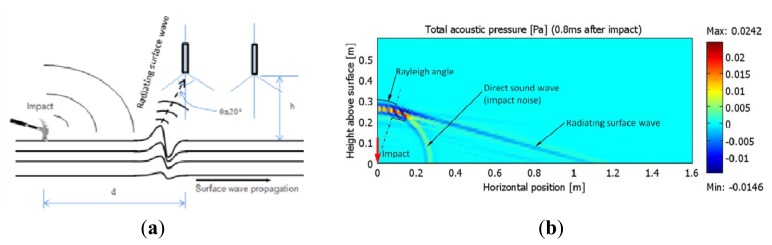
(**a**) Propagation of radiating surface wave. (**b**) Visualization of radiating surface wave by FEM.

Consider a layered pavement structure subjected to a vertical point impact at the surface, as shown in Figure 2(a). Besides the propagation of body waves, including P-waves and S-waves, the surface wave (Rayleigh wave) propagates horizontally near the free surface with dominant energy. Above the surface, the ground vibration due to the surface wave acts as an acoustic source to radiate an acoustic wave in the air. This acoustic radiation (radiating surface wave) from the ground surface wave brings the same information about the dispersion properties with frequency/wavelength. A visualization snapshot by FEM is presented in Figure 2(b) [19]. It indicates the dominant radiating surface wave arrives earlier than the direct sound wave caused by impact. According to the Snell's laws, the Rayleigh angle θ is determined by:

(1) $$\sin(\theta )=\frac{C_{a}}{C_{R}}$$

where, *C_a_* and *C_R_* are the speed of sound in the air and the surface wave speed in the pavement, respectively. The speed *C_a_* is approximately 340 m/s, and *C_R_* is approximately at the level of 1,000 m/s for many road constructions. These speeds lead to a radiation Rayleigh angle θ of approximately 20°. The radiating surface wave can be detected with directional microphones, which usually have an effective conical angle of around 100°. Aligning the angle of the directional microphones to match the angle of the radiating surface wave helps improve the signal to noise ratio of the measured acoustic data.

**Figure 3.**
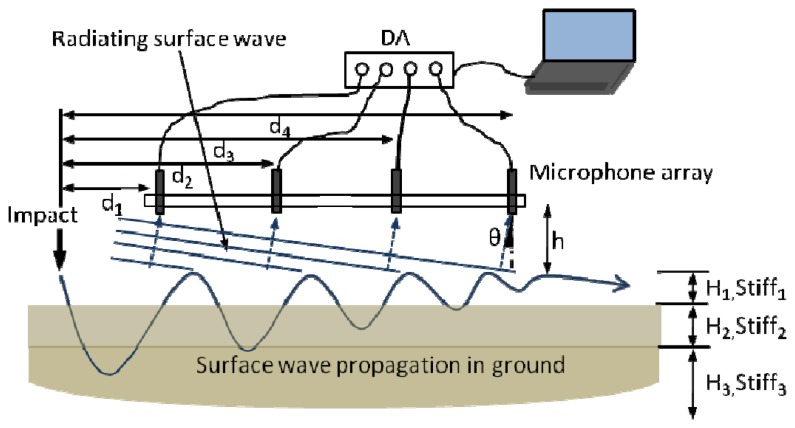
Air-coupled SASW configuration (like MASW with more channels).

By using acoustic radiation to sense the surface wave, the SASW test can be performed with microphones instead of accelerometers. Figure 3 shows a schematic configuration of the air-coupled SASW (similarly to the MASW) test. Two or more microphones are placed at a small distance above the ground and connected to the data acquisition device and computer. When a vertical impact is applied, the measured acoustic signal contains radiation from the surface wave (radiating surface wave), as well as the direct acoustic wave from the vibration in the vicinity of impact. The radiating surface wave brings the dispersion feature to indicate both the layer thickness as H_i_ and stiffness as Stiff_i_ in Figure 3. When the microphones are near the surface and the shear velocity of the pavement is much larger than the sound speed, the direct acoustic wave from the impact arrives to the microphone later than the radiating surface wave. The difference of their arrival time can be calculated as:

(2) $$\Delta t=\frac{\sqrt{d_{i}^{2}+h^{2}}}{C_{a}}-\frac{d_{i}-h\tan\theta }{C_{R}}-\frac{h}{C_{a}\cos\theta }$$

For a typical configuration with *d_1_* = 0.5 m and *h* = 0.05 m, the time delay is approximately 1 ms. Therefore, the radiating surface wave can be extracted from the intact acoustic signal by applying an appropriate time window.

**Figure 4.**
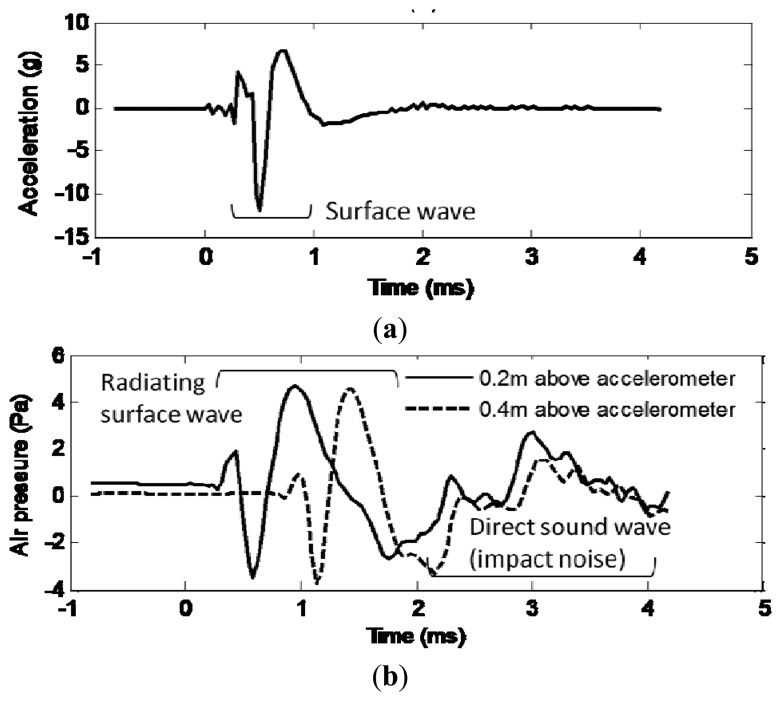
Comparison of microphone signal with accelerometer signal. (**a**) Measured surface acceleration. (**b**) Measured acoustic above surface signal.

Figure 4 shows an example of the surface wave measured by accelerometer (a) and the radiating surface wave measured by microphones (b). According to the comparison, it can be seen the first part of the microphone data arrives nearly at the same time and shows the similar feature as the accelerometer data. This part of data can be identified as the radiating surface wave. The second part of the microphone data is regarded as the direct sound wave from the hammer knocking because no signal is present in the acceleration signal counterpart at the same time.

Currently, some efforts have driven surface wave-based methods from traditional ground-contact sensing into non-contact sensing, either through an air-coupled transducer or an optical laser transducer. Earlier efforts like [20] sought to detect subsurface defects on bridge decks with acoustics. Recently, the authors of [16] described the air-coupling sensing theory and application. In their work, it was demonstrated that the acoustic radiation from the surface wave (radiating surface wave) could be acquired and processed similarly to the acceleration or velocity of the structure response. The replacement of ground-contact sensors with non-contact microphones would significantly improve the testing efficiency. However, there still exist difficulties to realize this efficient concept. One thing is the de-noising when the acoustic methods are used in the actual fieldwork subject to environmental noise. For this point, both the time windowing in the software and the de-noising in the hardware are applied in this paper. The time windowing in software was realized in an autonomous manner by using radiating surface wave arrival time estimation. The implementation of this automatic pre-processing to extract the radiating surface wave is introduced in the subsequent section.

### 2.2. Radiating Surface Wave Signal Extraction

Since the MASS system is designed with an orientation as an autonomous signal processing prototype, a pre-processing program implementing the automatic windowing for radiating surface wave is embedded in the software platform. The data acquisition is triggered by the impact acceleration through a professional shock accelerometer mounted coaxially on electromagnetic hammer core. The accelerometer monitors the hammer impact and triggers the data acquisition as the starting point in the time history. The time stamp where the first apparent peak or valley (max absolute amplitude) appears in the recorded acoustic data is identified as the arrival time of the radiating surface wave. A time-window with a Hanning shape is applied to truncate the signal of the radiating surface wave. The window center is aligned with the time stamp of the radiating surface wave, while the window size as arrival time difference from the radiating surface wave to the direct acoustic wave (Equation (2)). This windowing is realized automatically.

Additionally, several criteria are used in the pre-processing program to ensure acquired signal quality, especially in a field work environment. Firstly, the impact quality itself is verified through its time historical shape as acceleration, and only sharp impacts with broadband excitation would be accepted. Secondly, the magnitude squared coherence between sensing channels are also verified within 100 Hz to 10,000 Hz (Equation (3)):

(3) $$C_{xy}(f)=\frac{{\left|P_{xy}(f)\right|}^{2}}{P_{xx}(f)P_{yy}(f)}$$

Signals with low coherence in this range imply poor signal quality and would be neglected. Recorded signals that failed to pass the pro-processing criteria would be discarded for further processing. A message window would display to indicate more trials being needed. Only the acquired signals that satisfy the criteria are used as raw data for further processing. The raw data with poor quality due to random environment noise such as low frequency ground vibration would not be used for analysis.

### 2.3. De-Noising Hardware Design

**Figure 5.**
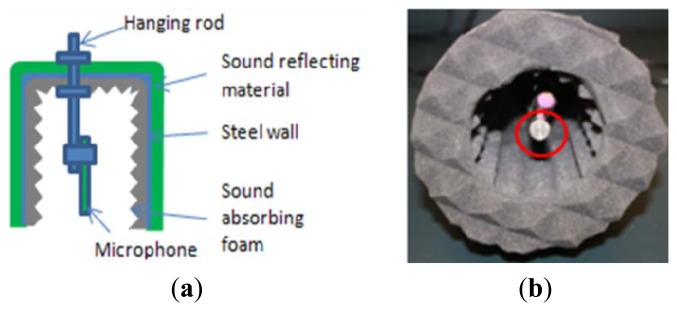
De-noise sound enclosure for microphone. (**a**) The schematic design of sound enclosure. (**b**) Built-up sound enclosure.

In order to improve the signal to noise ratio of acoustic data for the application in fieldwork, a sound barrier enclosure is designed to block the direct sound wave (impact noise) as well as the environmental noise. Figure 5 shows the design and a hardware realization. A steel cylinder is selected as the skeletal structure. A sound reflecting material with denser skin material wraps over the external surface of cylinder skeleton to reduce the penetration of outside noise. The acoustic noise would be mainly reflected on the denser skin surface first when it touches the skin. Then the noise penetrating the first reflection surface could be reflected by the steel cylinder wall again and attenuates in the form layer between the steel cylinder wall and the denser skin. A pyramid array pad is fitted in the internal surface of the skeleton and also the bottom circular surface to absorb reflections inside the chamber (Figure 5(b)). The microphone is mounted in the center of the cylinder with 1 cm above the ringed end plane as red circle marked in Figure 5(b).

Such a design would block the ambient noise and impact noise whose incident directions are almost perpendicular to the enclosure cylindrical surface. In contrast, the radiating surface wave from the ground vibration would easily reach the microphone (Figure 2(a)).

**Figure 6.**
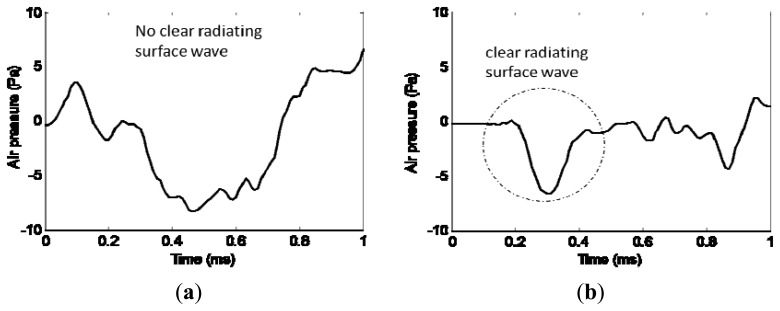
Effects of the sound enclosure for microphone in noisy environment. (**a**) Without enclosure. (**b**) With enclosure.

Figure 6 indicates the effect of this sound enclosure. The microphone is 0.2 m away from the impact source. The comparison is realized with two impacts, one without enclosure and one with enclosure. Figure 6(a) shows that without enclosure, the noise level is so high that the radiating surface wave can hardly be separated from the environment noise in recorded data. After the enclosure is applied to the microphone (Figure 6(b)), the radiating surface wave can be easily identified and extracted from the time history signal. Moreover, the enclosure blocks other ambient noise arriving before the radiating surface wave. As could be observed in Figure 6(b), the recorded acoustic signal before the radiating surface wave is satisfactorily close to zero. This indicates a high signal to noise ratio.

**Figure 7.**
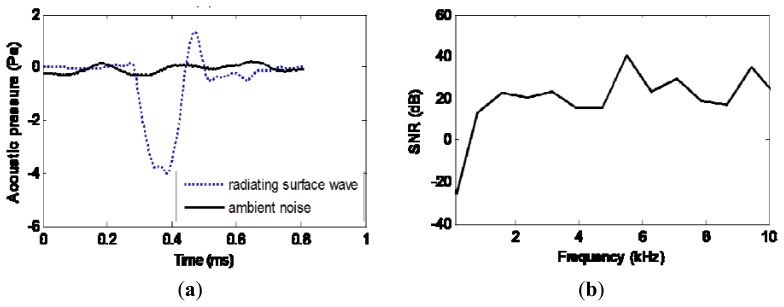
Ambient noise. (**a**) Comparison of extracted radiating surface wave and ambient noise. (**b**) Signal to noise ratio.

After the efforts as noted earlier to extract the radiating surface wave signal, the signal to noise ratio in the field work environment is studied using the Pingree Bridge Deck test (Section 5.3). As Figure 7(a) shows, the extracted radiating surface wave signal during impact is compared with the ambient noise during impact intervals. The average dB level for radiating surface wave is 96 dB, while the ambient noise contributes 75 dB. The signal to noise ratio is also indicated in Figure 7(b) within 10 kHz. The SNR is around 20 dB, except at the very low frequency end. This could be caused by the low frequency vibration of the bridge structure. However, this low frequency data would not affect the profile analysis for shallow depths within 1 m, since the corresponding penetration depth respect to wavelength is large.

## 3. MASS Hardware Platform Integration

**Figure 8.**
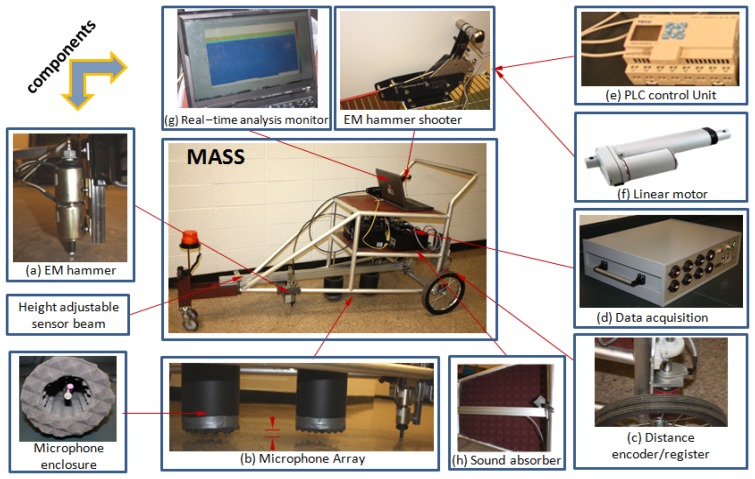
Integration of hardware of MASS.

The Mobile Acoustic Subsurface Sensing (MASS) system is constructed on a 3-wheeled cart. The cart platform is designed with an emphasis on stability during movement and impacting through vibration damping components. The MASS and its subsystems are presented in Figure 8. The first critical subsystem is the electronic hammer subsystem indicated as (a) in Figure 8. The hammer is based on two coaxial solenoids which lift and shoot the impact core. The impact properties could be controlled by tuning the power time on each solenoid with a time relay delay unit (528-TDRSOXP-24V). Due to the requirement of broad frequency band impact, the ideal impact would be close to a Dirac delta function. In order to monitor the impact quality and trigger the data acquisition, a professional shock accelerometer (PCB 350B03) is mounted on the impact core to watch the impact acceleration in real-time. Due to the variation in the roadway properties, the impact core is designed with replaceable tips with different stiffness.

The distance encoder/register subsystem (Figure 8(c)) is assembled with encoder mounted on the rear wheel and a PLC control unit (Figure 8(e)). The sensing spatial resolution is adjustable by changing the parameter setting at the PLC unit.

The MASS DAQ consists of a signal conditioner, a DAQ mother board, a Single Board Computer (SBC), DC power converter, and solid state hard drive. In the system, a SCM5B48 unit is selected as the signal conditioner. The PC104P-24DSI12 is selected as a 12-channel DAQ board which features low noise, 24-bit resolution, low phase distortion and multi-board synchronization. An embedded single board computer (SBC) called *Mamba* from Versalogic (Tualatin, OR, USA) with a high-performance Intel Core 2 Duo processor, computes at up to 2.26 GHz. The Operation System is QNX. The system is realized as multiple-channel and high sampling rate.

The microphone array is mounted as shown Figure 8(b), the array is isolated from the cart with professional dampers to isolate the vibration from the cart frame, especially while the impact is acting. The clearance from microphones to ground is adjustable so that the system is adaptable to different ground surface profiles.

## 4. Software Framework

The MASS software framework consists of three modules: (1) the data acquisition program under QNX operating system which sets up the sampling configuration; (2) a fast air-coupled SASW analysis program [18] under the MATLAB framework in the laptop; and (3) the real-time data communication between the DAQ and laptop.

**Scheme 1.**
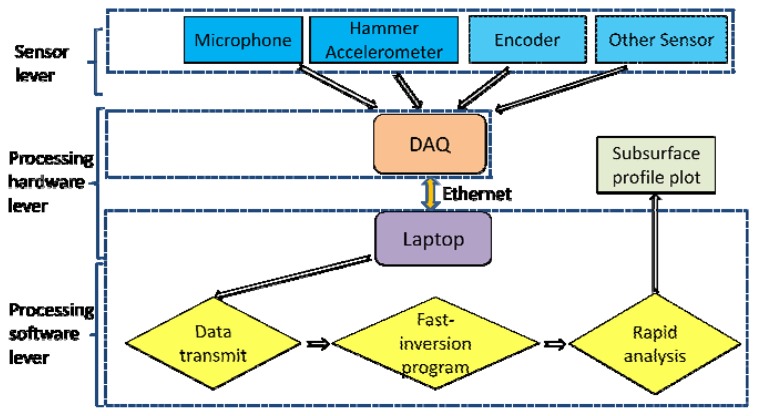
Hardware/software integration architecture.

One benefit of this design is that the convenient MATLAB programming environment is robust enough for the analysis program updating for the prototype development. Consequently, the architecture of hardware and software integration is shown is Scheme 1.

## 5. Implementation and Discussions

### 5.1. Asphalt Pavement

**Figure 9.**
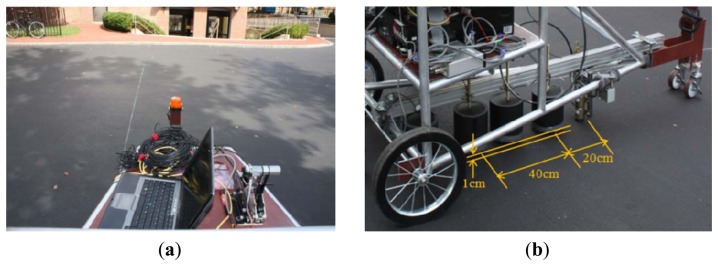
Remotion test on asphalt pavement. (**a**) Mobile sensing with MASS. (**b**) Test configuration.

The MASS system was tested on an asphalt pavement located near the Northeastern University campus. The testing path was along a white chalk line as shown in Figure 9(a). The testing configuration is shown in Figure 9(b), where the microphone enclosure is 1 cm above the ground while the near (channel A) and father (channel B) microphones are 20 cm and 60 cm apart from the impact source, respectively. The sampling rate is set as 200 kHz for all sensing channels such as the microphones and hammer accelerometer. The entire system could be operated by only one person at walking speed after set-up.

**Figure 10.**
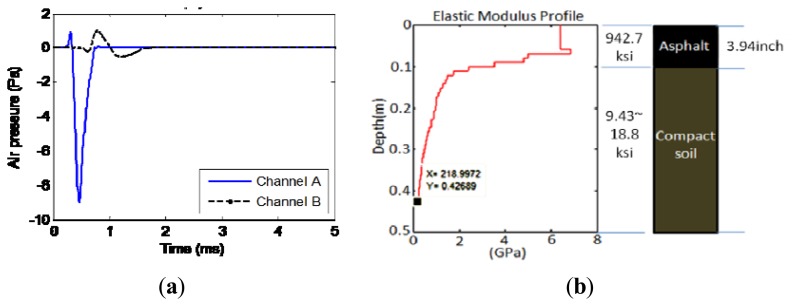
Testing on asphalt pavement. (**a**) Extracted radiating surface wave. (**b**) Estimated elastic modulus profile.

A typical testing point result is shown in Figure 10. After the pre-processing in software as mentioned in Section 2.2 and with the de-noising sound enclosure as mentioned in Section 2.3, the radiating surface wave is extracted. Figure 10(a) indicates that the extracted signals are clean and smooth. The inversion result to indicate the stiffness profile is shown in Figure 10(b). Without prior information about the paving material, a uniform mass density of 1,800 kg/m^3^ and a Poisson ratio of 0.3 are assumed in the calculation of the elastic modulus as general civil material properties. According to the profile, the first asphalt layer has an estimated depth of 0.1 m (3.94 inches) and estimated elastic modulus of 6.5 GPa (942.7 ksi), which falls into the range of a regular asphalt concrete material from 4.4 GPa (638.2 ksi) to 6.8 GPa (986.3 ksi). Also the thickness of the top layer matches with the typical roadway paving design. Underneath the top layer, the stiffness apparently decreases, as the modulus approaches 0.218 GPa (31.6 ksi) around 0.4 m (15.7 inches) depth. This is estimated to be compacted soil material.

### 5.2. Concrete Slab

**Figure 11.**
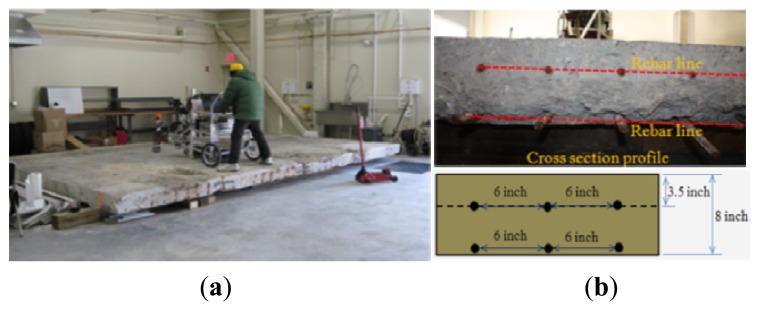
Concrete slab test configuration. (**a**) Test set-up. (**b**) Slab cross-section profile.

The MASS system was also tested on a concrete slab in the Laboratory for Structural Testing of Resilient and Sustainable Systems (STReSS Lab) at George J. Kostas Research Institute for Homeland Security at Northeastern University. The same test configurations as above are employed. Figure 11(a) records the test scenario, where the MASS cart is working in the central area of the slab in order to avoid the reflections of the stress waves due to the limited slab size. The cross-section of the slab is photographed as Figure 11(b), with a symbolic diagram underside. The slab is embedded with two rows of metal rebar located 8.9 cm (3.5 inches) below the surface for the top row while the bottom row is close to the bottom surface. The thickness of the slab is 20.32 cm (8 inches).

**Figure 12.**
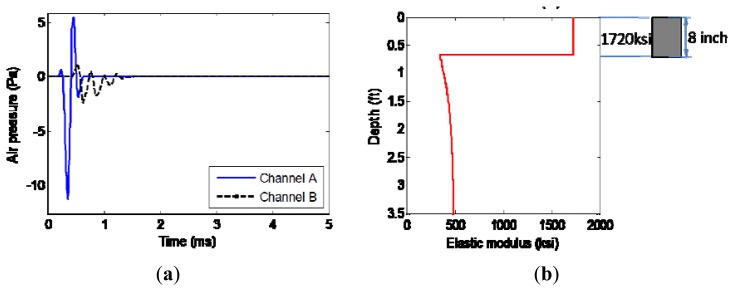
Concrete slab test results. (**a**) Extracted radiating surface wave. (**b**) Estimated elastic modulus profile.

The testing results are presented in Figure 12. The extracted radiating surface wave is presented in Figure 12(a), where the signals are clean enough for post-processing after pre-processing as mentioned in Sections 2.2 and 2.3. Comparing with the radiating surface wave extracted from the asphalt pavement shown in Figure 10(a), the signals coming from the stiff concrete slab present fluctuations with higher frequency. When the stiffness of the top layer is much larger than that of the sub-grades underneath, the surface wave approaches the Rayleigh-Lamb wave. Without using multiple-channel sensing to resolve the higher modes, the fast air-coupled SASW procedure with only two channels still offers an acceptable estimation of the profile as seen in Figure 12(b). The estimated total thickness of around 20.32 cm (0.67 ft) coincides accurately with the actual thickness (Figure 11(b)). The average elastic modulus of the concrete slab is estimated as 11.9 GPa (1,720 ksi). This modulus estimation yields the normal concrete slab modulus range and is a little on the low side. The minimum stiffness of 2.1 GPa (300 ksi) roughly deeper than the slab thickness could be caused by the wood beam foundation (Figure 11(a)). Due to the large stiffness contrast between concrete slab and the wood beam foundation, the deeper profile of the lab ground would not be identified from Figure 12(b). This test demonstrates the capability of the MASS system for concrete slab sensing in a floating style through acoustics.

### 5.3. Concrete Bridge Deck

**Figure 13.**
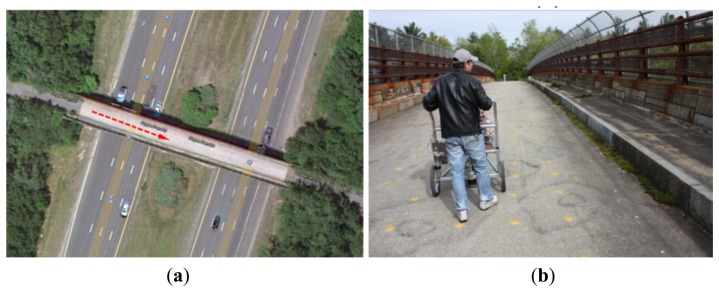
Pingree bridge deck test (Rowley, MA, USA). (**a**) Scanning path on bridge deck. (**b**) MASS operation on bridge deck.

Beside the capability for sensing the pavement structure, MASS system has the potential for bridge deck structure sensing. The fast air-coupled surface wave analysis program could be used for the bridge deck test with a Lamb wave inversion when the half-space foundation is deleted. The MASS system tests were performed on a bridge deck on the Pingree Bridge (Pingree Farm Rd, Rowley, MA, USA). The bridge consists of single layer concrete deck with as designed 9 inch thickness and spans around 240 ft above the Interstate 95 highway (Figure 13(a)).

**Figure 14.**
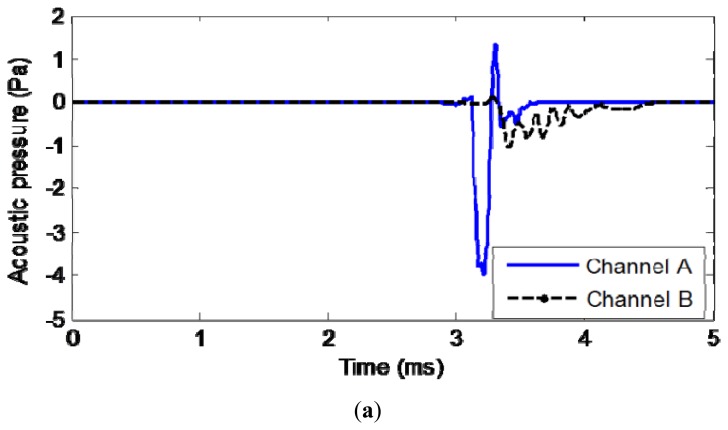

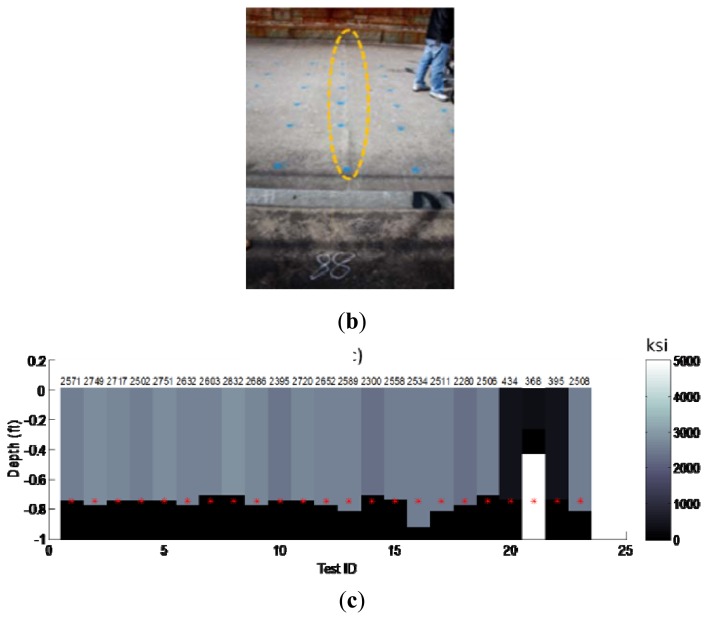
Pingree bridge deck test results. (**a**) Extracted radiating surface wave. (**b**) Joint crack. **(c)** Continuous scanning results.

The test is implemented in the real traffic environment with traffic noise. The MASS system configuration is maintained the same as in the preceding tests. The scanning is executed along a path (the red dotted line with arrow in Figure 13(a)) where no support beam exists underneath. The continuous scanning test results are presented in Figure 14. In total,m 23 test points are scanned with 1.22 m (4 ft) spacing along the scanning path. Figure 14(a) shows the filtered acoustic signals in the form of the extracted radiating surface wave. It could be observed that the signal features are close to those of the concrete slab as in the previous case. Higher frequency signal components are apparent. The successive profiles acquired through the mobile scanning at a walking speed are summarized in Figure 14(c). The results are consistent along the same path and the estimated thickness and close to the designed thickness at 0.23 m (0.75 ft) (red star line in Figure 14(c)). The averaged modulus of the deck is 17.7 GPa (2580 ksi). The profile at points 20^th^, 21^st^ and 22^nd^ give an abnormally low modulus around 2.7 GPa (400 ksi). This abnormity is caused by passing over the slab joint crack as shown in Figure 14(b). The slab joint crack can be identified sensitively. This fieldwork test verifies the potential for bridge deck testing using the MASS system, which is designed originally for pavement structures, when some modifications are made to the program.

## 6. Conclusions and Future Work

The transportation community has seen the great importance of technologies that can provide fast inspections of highways and bridge deck health assessments without interfering with or stopping traffic. In this paper, a mobile acoustic testing system is developed to estimate the subsurface profiles for both the thickness and stiffness of layers. The air-coupled non-contact sensing strategy is adopted in the MASS development. A fast air-coupled surface wave analysis program [18] is embedded to enhance the signal processing efficiency. The integration of the hardware utilizes the distance register/trigger together with an electronic hammer as impact source to supply repeatable and stable excitation. The de-noising sound enclosure and vibration isolation design improve the feasibility of acoustic sensing in actual fieldwork. The prototype hardware and software combination is demonstrated successfully. The system has been tested for asphalt pavement, a concrete slab and a concrete bridge deck. The reasonable matching of estimated results with exact subsurface profiles demonstrates the strength of the MASS system concept. Despite these achievements, more efforts are still underway. A better impact source with adjustable force and highly robust contact shapes for the test targets are under construction. Additionally, more efficient de-noising devices are also under research to supply cleaner and higher signal to noise ratios for acoustic sensing under heavy traffic noise conditions. Also, more field tests are planned to enhance MASS system's practical capability.
